# Magnetic Resonance Imaging Determined Visceral Fat Reduction Associates with Enhanced IL-10 Plasma Levels in Calorie Restricted Obese Subjects

**DOI:** 10.1371/journal.pone.0052774

**Published:** 2012-12-26

**Authors:** Gloria Formoso, Merilda Taraborrelli, Maria T. Guagnano, Monica D’Adamo, Natalia Di Pietro, Armando Tartaro, Agostino Consoli

**Affiliations:** 1 Department of Medicine and Aging, “G. d’Annunzio” University, Chieti-Pescara, Italy; 2 Department of Internal Medicine, University of Rome Tor Vergata, Rome, Italy; 3 Department of Experimental and Clinical Sciences, “G. d’Annunzio” University, Chieti-Pescara, Italy; 4 Department of Neuroscience and Imaging, “G. D’Annunzio” University, Chieti-Pescara, Italy; 5 Aging Research Center, Ce.S.I., “G. d’Annunzio” University Foundation, Chieti-Pescara, Italy; University of Tor Vergata, Italy

## Abstract

**Background:**

Obesity is characterized by a low grade chronic inflammation state. Indeed circulating pro-inflammatory cytokines, such as TNF-α and IL-6, are elevated in obese subjects, while anti-inflammatory cytokines, such as IL-10, appear to be reduced. Cytokines profile improves after weight loss, but how visceral or subcutaneous fat loss respectively affect pro- or anti-inflammatory cytokines plasma levels has not been precisely assessed. Therefore in the present study we correlated changes in circulating cytokine profile with quantitative changes in visceral and subcutaneous adipose tissue depots measured by an ad hoc Magnetic Resonance Imaging (MRI) protocol before and after weight loss.

**Materials and Methods:**

In 14 obese subjects**,** MRI determination of visceral and subcutaneous fat and plasma glucose, insulin, TNF-α IL-6, and IL-10 measurements were performed before and after a caloric restriction induced weight loss of at least 5% of the original body weight.

**Results:**

Weight loss improved insulin sensitivity (QUICKI Index: 0.35±0.03 vs 0.37±0.04; P<0.05), increased IL-10 (3.4±1.9 vs 4.6±1.0 pg/mL; P<0.03), and reduced TNF-α and IL-6 plasma levels (2.5±1.3 vs 1.6±1.5 pg/mL, P<0.0015, 2.3±0.4 vs 1.6±0.6 pg/mL, P<0.02 respectively). A significant correlation was observed between the amount of visceral fat loss and the percentage reduction in both TNF-α (r = 0.56, p<0.05) and IL-6 (r = 0.19 p<0.05) plasma levels. In a multiple regression analysis, the amount of visceral fat loss independently correlated with the increase in IL-10 plasma levels.

**Conclusion:**

The reduction in visceral adipose tissue is the main driver of the improved inflammatory profile induced by weight loss.

## Introduction

Adipose tissue synthesizes and releases into the circulation a large number of adipokines, most likely contributing, in obese individuals, to vascular inflammation, endothelial dysfunction and atherosclerosis and participating in the regulation of metabolic processes [1]. Indeed, circulating pro-inflammatory cytokines, such as Tumor necrosis factor (TNF)-α and interleukine-6 (IL-6), are elevated in obese subjects [2], while plasma levels of anti-inflammatory cytokines, such as interleukine-10 (IL-10), appear to be reduced [3]. Diet-induced weight loss is accompanied by an inflammatory cytokines decrease [4], [5] and by an IL-10 increase in plasma [6].

Obesity, however, remains a puzzling condition for clinicians, due to its remarkable heterogeneity, mainly related to differences in the adipose tissue regional distribution [7]. Indeed it appears that intra abdominal fat accumulation is far more detrimental than subcutaneous fat deposition as it pertains to metabolic derangement [8] and cardiovascular risk [9]. Several inflammatory cytokines are definitely more expressed in visceral than in subcutaneous adipose tissue [10]: one could thus speculate that visceral fat accumulation is mainly responsible for the altered cytokine profile in obesity. This, however, has not been definitely proven. Indeed, little is known about how changes in visceral and/or subcutaneous fat tissue might affect plasma levels of pro-inflammatory or anti-inflammatory cytokines and this has not been through fully investigated in intervention studies employing reliable techniques for the assessment of visceral and subcutaneous fat mass changes respectively. Aim of the present study was therefore to measure by an ad hoc Magnetic Resonance Imaging protocol quantitative changes in visceral and subcutaneous adipose tissue depots induced by caloric restriction and to correlate these with changes in circulating cytokine profile.

## Methods

### Subjects

Twenty-one subjects (BMI range 28–41 kg/m^2^) whose anthropometric characteristics are summarized in Table 1 were recruited at Pescara Town Hospital University Diabetes Clinic (Pescara, Italy) and at Chieti Town Hospital University Obesity Center (Chieti, Italy).

**Table 1:** Clinical and anthropometric parameters of study population.

	Baseline	After weight loss≥5%	*p
N	14	14	
Age (years)	42±14	42±14	
Sex (M/F)	4/10	4/10	
Waist Circumference (cm)	104±15	98±16	<0.01
Weight (Kg)	88±10	82±10	<0.01
BMI (Kg/m^2^)	34±6	32±6	<0.01
SBP (mmHg)	137±4	133±7	n.s.
DBP (mmHg)	85±5	85±6	n.s.

Data are expressed as mean±SD. BMI: Body Mass Index; SBP: Systolic Blood Pressure; DBP: Diastolic Blood Pressure; n.s.: not significant.

All subjects were sedentary (<20 min of aerobic exercise twice a week), non-smoker and normotensive. They did not present any disease of major organs or systems nor any major alteration of blood chemistry, lipid profile or urinary albumin excretion.

### Protocol

The study protocol was approved by the University G. D’Annunzio Ethics Committee and conducted according to the Helsinki Conference principles.

Patients who had signed a written informed consent form reported to clinic in the morning, after an overnight fast. Anthropometrical measurements were taken by a trained staff according to the World Health Organization recommendations [11]. All subjects underwent a 120 minutes oral glucose tolerance test (OGTT) with 75 g glucose to exclude diabetic state. During OGTT, blood samples were obtained at time 0; 60; 120.

On each sample, plasma glucose and insulin were determined. On samples drawn at time 0, lipid profile, and plasma IL-10, TNFα, IL-6 and Leptin levels were also determined. Evaluation of insulin sensitivity was performed by QUICKI INDEX calculation using fasting insulin and glucose concentration [12]. An MRI of the abdominal region was then performed for quantification of visceral and subcutaneous fat distribution [13] and patients were entered in a counselling program devised to induce weight loss by caloric restriction (average caloric deficit = 500 Kcal/day) and increased physical activity (patients were encouraged to engage in a 45 min fitness walk 4 times a week which had to be recorded in a log book). The mean recommended daily caloric intake was 1.300 kcal, ranging from 1.250 to 1.350 kcal. The recommended composition of the dietary regimen was 55–60% carbohydrate, 15–20% protein, and 20–25% fat. Subjects reported to control visits every two weeks. When a whole body weight loss equal to 5% of the initial body weight was achieved, patients were tested again as they had been at baseline and MRI quantification of visceral and subcutaneous fat was repeated.

### Clinical Laboratory Measurements

Routine chemical analysis were assessed in the hospital’s chemistry laboratory. Serum samples for TNF-α, IL-6, Leptin and IL-10 were stored at −80°C and were assayed in duplicate using an high sensitivity, quantitative sandwich enzyme assay (Quantikine HS, R&D System, Inc., Minneapolis, MN).

### MRI Quantification of Visceral and Subcutaneous Fat

MRI examination was performed by using a whole body scanner (Magnetom, Siemens), operating at 1.5 Tesla, equipped with a dedicated transmit/receive body coil. Scan protocol included a preliminary scout view sequence acquired on median sagittal plane by using a gradient echo 2D turbo FLASH sequence (3 slices 10 mm tick, TR 30 ms, TE 5 ms, FA 65°). A 2D fast low*-*angle shot *(*FLASH*)* T1-weighted breath-hold sequence was acquired on the axial plane. Scan parameters were: twenty one slices 6 mm thick positioned on L3 vertebral body (TR 40 ms; TE 6 ms; FA 55°).

Image analysis was performed on a dedicated work-station using a semi-automatic quantitative segmentation method. Subcutaneous and visceral adipose tissue were divided by drawing a line along the internal border of the thoracic, abdominal and back muscles. Visceral and subcutaneous fat were then labeled by assigning them different color codes. The adipose tissue areas were finally computed automatically by summing adipose tissue pixels and multiplying by the pixels surface area [14].

### Data Analysis

Results are expressed as mean±SD or as interquartile ranges (as in the case of cytokines values). Differences were assessed by Student *t* test or Wilcoxon rank test when appropriate. Correlations between parameters were assessed using bivariate correlation analysis (Pearson correlation coefficient). Multivariate linear regression analysis performed by stepwise variable selection was used with changes in TNF-α, IL-6 and IL-10 as dependent variables. Covariate testing included BMI, body weight and waist circumference to asses relationship among variables. Significance was defined as *P*<0.05. All analyses were performed using SPSS software program Version 12.0 for Windows.

## Results

### Baseline Characteristics

Fourteen subjects, (4 male, 10 female) successfully complied with the weight loss program and ended up loosing 5% or more of the initial body weight. Characteristics of these subjects and baseline anthropometric as well as biological parameters are shown in table 1 and 2.

**Table 2:** Metabolic parameters of study population.

	Baseline	After weight loss≥5%	*p
FPG (mmol/L)	4.72±0.61	4.50±0.39	n.s.
Plasma insulin(pmol/L)	15.00±9.00	10.00±4.00	<0.05
Total Cholesterol (mmol/L)	5.40±1.21	4.62±0.85	<0.05
Triglyceride (mmol/L)	1.10±0.65	0.98±0.56	<0.05
HDL (mmol/L)	1.34±0.26	1.55±0.36	<0.01
LDL (mmol/L)	3.46±1.39	2.81±0.67	n.s.
QUICKI Index	0.35±0.03	0.37±0.04	<0.05
Leptin (ng/mL)	56.10±30.20	37.20±29.30	<0.05

FPG: fasting plasma glucose. Data are expressed as mean±SD; n.s.: not significant.

### Anthropometric Parameters

As shown in table 1, caloric restriction determined a significant weight loss (88±10 vs 82±10 Kg; P<0.01), with an average weight reduction of 6.2±1.8 Kg accomplished in the average time of 8 months. BMI dropped from 34±6 Kg/m^2^ to 32±6 Kg/m^2^ (P<0.01) and this was accompanied by a reduction in waist circumference from cm. 104±15 to cm 98±16 (P<0.01). No correlation was observed between waist circumference reduction and visceral fat loss (both expressed as percent of basal values, r = −0.034, P = 0.91).

### Glucose, Lipids and Insulin Sensitivity

Following weight loss, total cholesterol and triglyceride levels significantly decreased (5.40±1.21 vs 4.62±0.85 and 1.1±0.65 vs 0.98±0.56 mmol/L respectively; P<0.05 for both), HDL-cholesterol significantly increased (1.34±0.26 vs 1.55±0.36 mmol/L; P<0.01) while LDL-cholesterol showed a trend toward a decrease which did not reach, however, statistical significance (3.46±1.39 vs 2.81±0.67 mmol/L; P = ns) (table 2).

As shown in table 2, plasma insulin levels decreased after weight loss (15±9 vs 10±4 pmol/L; P<0.05), while insulin sensitivity, evaluated by the QUICKI index, improved (0.35±0.03 vs 0.37±0.04; P<0.05). Leptin levels were significantly reduced (56.1±30.2 vs 37.2±29.3 ng/mL P<0.05). No changes were observed in fasting plasma glucose (4.72±0.61 vs 4.50±0.39 mmol/L P = ns) nor in the glucose area under the curve after OGTT.

### Inflammatory and Anti-inflammatory Cytokines

**Figure 1:**
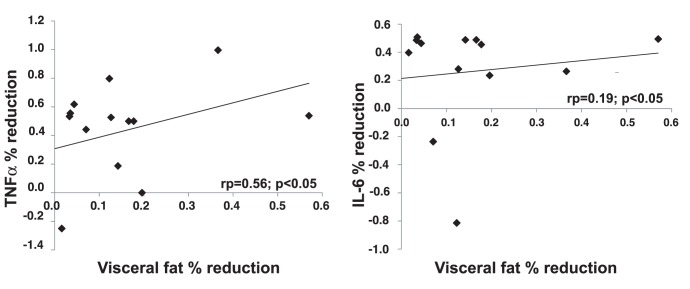
Correlation between percent visceral fat reduction and percent plasma IL-6 and TNF-α levels reduction in the study population.

**Figure 2:**
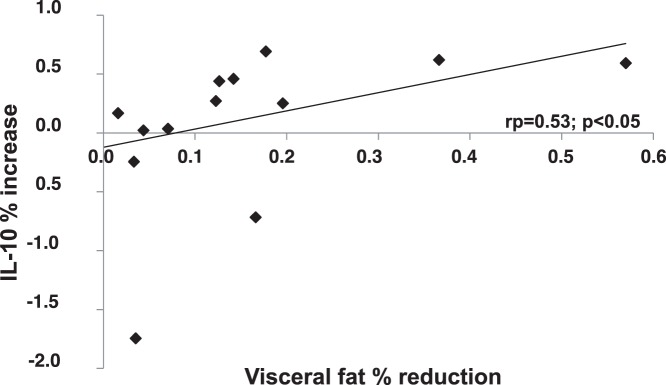
Correlation between percent visceral fat reduction and percent plasma IL-10 levels increase in the study population.

As shown in table 3, IL-10 plasma levels significantly increased after weight loss (3.4±1.98 vs 4.6±3.0 pg/mL; P<0.03) while, TNF-α and IL-6 levels were significantly reduced (2.5±1.3 vs 1.6±1.5 pg/mL, P<0.0015, 2.3±0.4 vs 1.6±0.6 pg/mL, P<0.02 respectively). A significant and positive correlation was observed between the amount of visceral fat loss and the improvement in insulin sensitivity (r = 0.04, P<0.05) and between the percentage reduction in visceral fat and the percent reduction in both TNF-α (r = 0.56; P<0.05) and IL-6 (r = 0.19; P<0.05) (Fig. 1). On the contrary no correlation was observed between the investigated parameters and either the amount or the percentage of subcutaneous fat lost. Interestingly, a positive correlation was also observed between the percentage of visceral fat loss and the percentage increase in IL-10 levels (r = 0.53; P<0.05) (Fig. 2). Furthermore, in a multiple regression analysis, the amount of visceral fat loss independently correlated with the increase in IL-10 levels (Table 4).

**Table 3:** Inflammatory parameters of study population.

	N	Mean	Std Dev	Median	Lower Quartile	Upper Quartile	*p
TNF-α basal	14	2.52	1.32	1.80	1.60	3.78	
TNF-α after weight loss	14	1.60	1.52	0.90	0.70	2.11	<0.0015
IL-6 basal	14	2.32	0.42	2.38	2.02	2.67	
IL-6 after weight loss	14	1.64	0.64	1.38	1.33	1.50	<0.02
IL-10 basal	14	3.41	1.98	3.11	2.37	4.62	
IL-10 after weight loss	14	4.63	3.03	3.73	2.85	7.44	<0.03

Data are reported as pg/mL.

**Table 4:** Multivariate regression analysis.

	Visceral fat variation	Subcutaneous fat variation	Body Weight variation	BMIvariation	Waist Circum.variation
IL-6	0.23P = 0.43	−0.17P = 0.56	0.04P = 0.89	−0.32P = 0.28	−0.25P = 0.40
TNF-α	0.14p = 0.63	0.31P = 0.28	−0.31P = 0.28	0.08P = 0.80	0.05P = 0.86
IL-10	**−0.53** **P = 0.05**	0.07P = 0.82	0.18P = 0.55	0.17P = 0.58	−0.22P = 0.45

Value in bold was statistically significant.

## Discussion

In the present study we used Magnetic Nuclear Resonance to measure visceral and subcutaneous fat depot before and after weight reduction, in order to establish whether changes in inflammatory cytokines pattern and in insulin sensitivity were more directly correlated to adipose tissue loss in one or in the other compartment.

As expected, following weight loss we observed improved insulin sensitivity, reduced TNF-α, IL-6 and leptin levels and increased IL-10 levels. When we set out to discern whether these improvements were due to a selective tissue loss from the visceral or the subcutaneous fat depot, we observed a significant correlation between the amount of visceral fat loss and the improvement in insulin sensitivity. We also found that the percentage reduction in visceral fat loss was significantly correlated with the percentage reduction in both TNFα and IL-6 plasma levels and with the percentage increase in IL-10 levels. On the contrary, no correlation was found between the investigated parameter and either the amount or the percentage of subcutaneous fat loss. Furthermore, in a multiple regression analysis, the amount of visceral fat loss independently correlated with the increase in IL-10 levels. To our knowledge, our data are the first to show that diet induced changes in visceral fat, measured by an objective and reliable technique, are definitely associated with changes in metabolic and inflammatory parameters while changes in subcutaneous fat are not.

Noteworthy, changes in visceral fat did not strictly correlate with changes in waist circumference: this stresses once more the concept that waist circumference could be used as a proxy of visceral fat accumulation only in large population studies but it is a much too imprecise measurement to be used to detect subtle changes in visceral fat amount [15], [16]. The fact that we did not have a control group could be perceived as a limitation of the study. This was mainly due to the fact that all of the subjects whose data were available did achieve a 5% reduction in body weight in less than one year (which we had set as the maximal follow-up length). However, since each subject was his own control, this should not affect the main results of the study.

The inflammatory state characterizing obesity is thought to be due to increased secretion of inflammatory cytokines by the adipocytes or by macrophages recruited into the expanding adipose tissue and differentiated into adipocytes [17]. This is supposed to happen particularly in the visceral adipose tissue [18]. Ample evidence indicates indeed that the regional distribution of adipose tissue is a key factor explaining the relationship between adiposity and cardiometabolic risk. Thus in an Asian cohort, visceral fat (measured by CT scan) but not subcutaneous fat progressively increased with increasing degree of glucose intolerance and was associated with increased levels of TNF-α [8]. In cross sectional studies, plasma levels of different pro-inflammatory cytokines have been found associated with visceral fat content [1]. Thus Cartier et al. in a cohort of 200 subjects found that plasma C reactive protein, TNF-α and IL-6 levels were all correlated with visceral fat content as measured by CT scan [19]. In keeping with this finding, Fontana et al. reported that IL-6 concentrations was 50% greater in the portal vein than in the radial artery in 25 extremely obese subjects undergoing gastric by-pass [20]. If the bulk of obesity related metabolic abnormalities is indeed related to the low grade chronic inflammation state brought about by an altered adipokines secretion pattern, then adipose tissue accumulation in the visceral district should be more directly related to such abnormalities as compared to adipose tissue accumulation in the subcutaneous district. This is supported by several animal and humans studies showing that visceral adipose reduction correlates with improvement in glucose and lipid metabolism. Surgical removal of visceral fat prevented the age-related insulin sensitivity impairment in the rat [21]. In obese subjects, omentectomy associated with adjustable gastric banding induced, for the same weight loss, a greater improvement in fasting plasma glucose, insulin sensitivity and glucose tolerance as compared to adjustable gastric banding alone [22], while removal of subcutaneous fat tissue by liposuction did not result in any metabolic benefit [23].

Much less information is however available as to whether visceral fat reduction is indeed associated with an improved adipokines profile far more than subcutaneous fat reduction. In mice, a reduction in abdominal adiposity obtained by bariatric surgery was accompanied by an improvement in adipose tissue inflammation [24]. In white women Fisher et al. found that caloric restriction induced weight loss resulted in a greater TNF-α decrease as compared to Afro American women and that this could be explained by a greater loss of intra abdominal adipose tissue as measured by dual-energy X-ray absorptiometry [25]. Our study is the first in which visceral and subcutaneous fat loss are directly compared in terms of association with changes in adipokynes profile. Our data do demonstrate that visceral fat loss is the main driver of the reduction in circulating TNF-α and IL-6 induced by weight loss.

Our results also show that visceral fat (but not subcutaneous fat) reduction was associated with an increase in IL-10 circulating levels. IL-10 is considered an anti-inflammatory cytokine with a potentially protective actions against development of both endothelial dysfunction and atherosclerosis [5]. Increased IL-10 circulating levels are thus emerging as a potential protective factor. As a matter of fact, IL-10 has been found decreased in obesity, metabolic syndrome and type 2 diabetes [3], [26]. In several, although not all, previous studies investigating the relationships between weight loss and adipokines pattern, caloric restriction and weight loss have been reported to increase circulating IL-10 levels. However, no study to our knowledge has so far investigated the relationships between IL-10 levels and visceral and/or subcutaneous fat loss. Our data are then the first demonstrating that visceral fat loss is also the main driver of an increase in this potentially important protective cytokine.

In conclusion, our data provide a convincing demonstration, based on the use of state of the art MRI techniques, that visceral fat loss, rather than subcutaneous fat loss, is associated with a significant improvement in the inflammatory cytokines profile in obese subjects put on a caloric restriction diet. Our study also show for the first time that visceral fat loss is significantly associated with an increase in IL-10 circulating levels. Taken together these results lend further meaningful support the concept that visceral fat is the main culprit for metabolic derangement in obesity and might provide further impulse for research aimed at finding new therapeutic strategies finalized to reducing visceral fat accumulation.
