# Correction: Magnetic Resonance Imaging Determined Visceral Fat Reduction Associates with Enhanced IL-10 Plasma Levels in Calorie Restricted Obese Subjects

**DOI:** 10.1371/annotation/53374aad-bc04-4c64-b313-8ff9b881d0da

**Published:** 2013-09-20

**Authors:** Gloria Formoso, Merilda Taraborrelli, Maria T. Guagnano, Monica D’Adamo, Natalia Di Pietro, Armando Tartaro, Agostino Consoli

There were multiple errors in the affiliations. The correct affiliations for the first, fifth, and seventh authors are:

Gloria Formoso

Department of Medicine and Aging, "G. d'Annunzio" University, Chieti-Pescara, Italy

Aging Research Center, Ce.S.I., "G. d'Annunzio" University Foundation, Chieti-Pescara, Italy

Natalia Di Pietro

Department of Experimental and Clinical Sciences, "G. d'Annunzio" University, Chieti-Pescara, Italy

Aging Research Center, Ce.S.I., "G. d'Annunzio" University Foundation, Chieti-Pescara, Italy

Agostino Consoli

Department of Medicine and Aging, "G. d'Annunzio" University, Chieti-Pescara, Italy

Aging Research Center, Ce.S.I., "G. d'Annunzio" University Foundation, Chieti-Pescara, Italy

Reference 11 should be reported as:

World Health Organization (1997) Obesity: Preventing and Managing the Global Epidemic. Report of a WHO Consultation on Obesity. . WHO/NUT/NCD/98I Geneva: World Health Organization. 

